# Simultaneous Liquid Digestate Treatment and High-Value Microalgal Biomass Production: Influence of Post-Harvest Storage on Biochemical Profiles

**DOI:** 10.3390/molecules30132778

**Published:** 2025-06-27

**Authors:** Ewelina Sobolewska, Michał Komar, Sebastian Borowski, Paulina Nowicka-Krawczyk, António Portugal, Nuno Mesquita, Mariana F. G. Assunção, Berk Aksoy, João Cotas, Leonel Pereira

**Affiliations:** 1 Department of Environmental Biotechnology, Faculty of Biotechnology and Food Sciences, Lodz University of Technology, Wólczańska 171/173, 90-530 Lodz, Poland; michal.komar@dokt.p.lodz.pl (M.K.); sebastian.borowski@p.lodz.pl (S.B.); 2 Interdisciplinary Doctoral School, Lodz University of Technology, Żeromskiego 116, 90-924 Lodz, Poland; 3 Department of Algology and Mycology, Faculty of Biology and Environmental Protection, University of Lodz, Banacha 12/16, 90-237 Lodz, Poland; paulina.nowicka@biol.uni.lodz.pl; 4 Centre for Functional Ecology (CFE)—Science for People & the Planet, Department of Life Sciences, University of Coimbra, Calçada Martim de Freitas, 3000-456 Coimbra, Portugal; aportuga@bot.uc.pt (A.P.); inunomesquita@gmail.com (N.M.); 5 TERRA—Associate Laboratory for Sustainable Land Use and Ecosystem Services, Department of Life Sciences, University of Coimbra, Calçada Martim de Freitas, 3000-456 Coimbra, Portugal; leonel.pereira@uc.pt; 6 FitoLab—Laboratory for Phytopathology, Instituto Pedro Nunes, Rua Pedro Nunes, 3030-199 Coimbra, Portugal; 7 Coimbra Collection of Algae (ACOI), Department of Life Sciences, University of Coimbra, 3000-456 Coimbra, Portugal; mariana.assuncao@uc.pt (M.F.G.A.); berk.aksy00@gmail.com (B.A.); 8 Marine Resources, Conservation and Technology, Marine Algae Lab, Centre for Functional Ecology (CFE)—Science for People & the Planet, Department of Life Sciences, University of Coimbra, 3000-456 Coimbra, Portugal; jcotas@gmail.com

**Keywords:** anaerobic digestion effluent, microalgae cultivation, photobioreactor, resource recovery, biomass storage, waste-to-value

## Abstract

This study investigated the treatment of unsterilized, undiluted, and unfiltered liquid digestate in a large-scale photobioreactor over a period of 33 weeks using a consortium of microalgae and bacteria. The generated biomass was analyzed for a wide spectrum of value-added compounds. The impact of organic loading rates (OLR) on the microbial culture was determined, and the influence of the biomass storage method on its qualitative composition was also analyzed. The experiment showed optimal growth of microalgae at OLR = 0.1 gCOD/L/day (where COD is Chemical Oxygen Demand), while a higher OLR value led to culture destabilization. *Microglena* sp., an algae not commonly applied for digestate treatment, showed low tolerance to changes in process conditions (OLR increase) but high readaptation potential when the OLR was lowered to its initial value. Significant changes in the microbial community were observed during the treatment. In Phases 1 and 2, *Desmodesmus subspicatus* and Actinomycetota phylum dominated in the community, while in Phase 3, *Microglena* sp. and Firmicutes were the most abundant. Total nitrogen, orthophosphates, and soluble COD were reduced by 89–99%. The biomass storage method had a notable impact on the content of lipids, fatty acids, and pigments. The protein amount was 32.75–33.59% of total solids (TS), while total lipid content was 15.76–19.00% TS, with stearic and palmitic acid being dominant. The effect of the storage regime on the potential biomass valorization was also discussed.

## 1. Introduction

As a result of the energy transition policy, bioenergy generated from plant and animal wastes [1] has become an integral part of the global energy economy. For the European bioenergy sector, utilization of biomass is essential, with anaerobic digestion now considered to be the main route for converting waste into valuable biogas [2]. Such a strategy is consistent with the sustainable development policy and can significantly contribute to waste recycling, the generation of clean energy, and combating ongoing climate change [3]. Despite the evident advantages, it is often overlooked that anaerobic digestion (AD) also produces huge amounts of by-product, mainly digestate [4]. With the growing number of biogas plants in Europe, the problems with their utilization are increasing. As a consequence, initiatives such as Vision 2020 and The Waste and Resources Action Program (WRAP, UK) have placed greater emphasis on the management of post-digestion effluents [5].

The digestate produced during AD consists of liquid and solid fractions, which differ in composition, properties, bioavailability, and means of direct application [4]. The most attractive way to use digestate is for agricultural purposes. However, this solution is limited by several difficulties currently obstructing green and circular biogas production technology. Untreated digestate can create environmental hazards, contribute to greenhouse gas emissions (especially during storage), leaching of nutrients, and is often uneconomical due to high transport costs [6]. Liquid fraction constitutes 80–90% of digestate and is considered more difficult to manage and process. Treatment strategies require significant energy input and can lead to the further formation of unfavorable by-products [7]. 

In recent years, the use of liquid digestate as a medium for the growth of microalgae has gained more interest. This technology can effectively utilize anaerobic digestion effluents, enables the recovery of nutrients, and generates valuable algal biomass. On the other hand, its efficiency depends on the microalgae strains used, as well as the origin and composition of the digestate [8]. The critical factors are the heterogeneity and variable nature of the liquid, high turbidity, dark color, unbalanced concentration of organic substances, ammonia toxicity, and sometimes the presence of heavy metals or bacterial metabolites [4,6,9]. In response to the above, the following strategies are used: selection of microalgal strains capable of growing in the liquid digestate, dosing or diluting the substrate to reduce color and elevated ammonia concentrations, and mitigating high levels of suspended solids and turbidity through filtration or centrifugation [10,11]. 

Nowadays, algal-derived biomass offers a promising material rich in lipids, fatty acids, pigments, proteins, amino acids, and compounds with antimicrobial activity [12]. The ability of photoautotrophic cells to accumulate significant amounts of lipids with an attractive composition enables the production of oils, and the accumulated fatty acids can also be used for biodiesel production. On the other hand, biomass can also be used as a biofertilizer or biostimulant, and algal post-extraction debris can be reused for biogas production via anaerobic digestion [13]. Compared to other compounds, pigments are produced with relatively low efficiency. However, due to their high bioactive and nutritional value, they significantly contribute to the profitability of refineries [14,15]. Algal biomass can also act as a protein source and be potentially used as a feed and food additive [16].

The appropriate use of microalgae may therefore be a key factor in the sustainable management of post-digestion effluents and the simultaneous generation of biomass with high biotechnological potential.

The aim of this study was to evaluate the long-term treatment of liquid digestate in a large-scale photobioreactor operated under variable organic loading rates. Contrary to most literature reports, the liquid digestate was not pretreated by filtration, dilution, or sterilization, as these operations increase the costs and complexity of the process. Only environmental isolates of microalgae were used for reactor inoculation, including *Microglena* sp. (Chlorophyta), which has so far only been applied in limited, small-scale studies [17,18]. The harvested biomass was then subjected to a detailed quantitative and qualitative analysis, which included lipids, fatty acids, proteins, and pigments determination. The impact of microalgal biomass storage on its composition was also analyzed.

It was hypothesized that long-term treatment of raw liquid digestate in a large-scale photobioreactor inoculated exclusively with environmental microalgal isolates (including *Microglena* sp.) would enable efficient removal of nutrients and organics while producing high-value algal biomass. It was also expected that the harvested biomass would exhibit favorable quantitative and qualitative characteristics, and that storage conditions would affect its biochemical composition, determining further applications.

## 2. Results and Discussion

### 2.1. Characteristics of Raw Liquid Digestate

The composition of the raw liquid digestate used in the experiment is shown in Table 1. The substrate was characterized by a pH close to neutral, which is preferred by most freshwater microalgal species [19]. The values of TSS (total suspended solids) (7.08 ± 0.67 mg/L) and turbidity (108.42 ± 2.47 FAU) were generally low compared to values reported in the literature [20], and suitable for algal cultivation. Both TSS and turbidity values are crucial for light transmittance, which strongly affects algal growth [9,10,21,22]. In contrast, the concentration of organics in the digestate, expressed as chemical oxygen demand (COD), was relatively high (total COD = 7414.17 ± 39.65 mgO_2_/L; soluble COD = 6411.67 ± 62.50 mgO_2_/L), while TN (total nitrogen) was within the range reported in the literature [7]. The liquid was also rich in orthophosphates (491.67 ± 26.23 mg/L), most likely resulting from the use of coagulants at the wastewater treatment plant, commonly applied to remove phosphorus and enhance dewatering processes. To reduce high concentrations of nutrients and organic contaminants and to avoid the impact of external bacterial microflora, liquid digestate is often subjected to preliminary treatment [7,13]. However, these operations (sterilization, dilution, discoloration) generate costs and increase installation complexity [23,24]. For this reason, no pretreatment was applied in this study. 

### 2.2. Photobioreactor Performance

**Figure 1:**
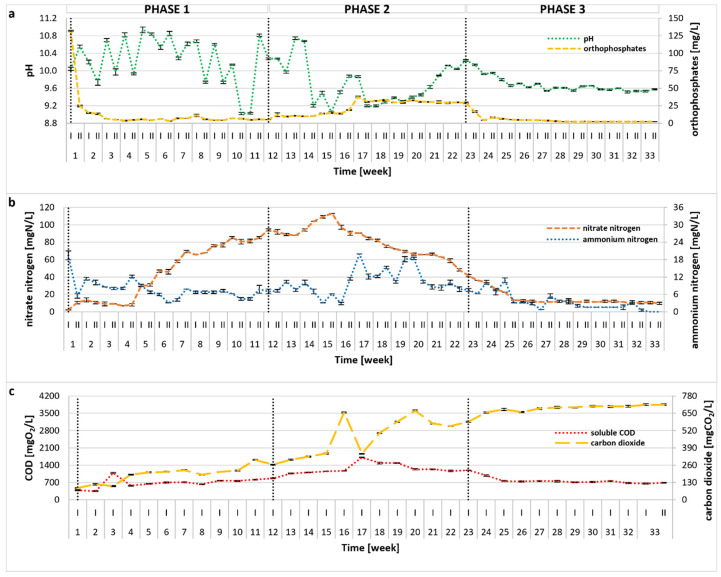
Changes in pH, PO_4_^3−^ (**a**), NO_3_^−^, NH_4_^+^ (**b**), sCOD, and CO_2_ (**c**) during the 33-week photobioreactor operation under different OLRs (Phase 1: 0.1 gCOD/L/d; Phase 2: 0.15 gCOD/L/d; Phase 3: 0.1 gCOD/L/d).

The photobioreactor was continuously operated for 33 weeks, and variations of the main process indicators (i.e., pH, PO_4_^3−^, NH_4_^+^, NO_3_^−^, sCOD, CO_2_) are plotted in Figure 1. Statistical comparisons between experimental phases are presented in Table S1. In the initial stage of the treatment, NH_4_^+^ remained at a relatively low level with only a slight drop during Phase 1, whereas the concentration of NO_3_^−^ showed a visible upward trend, suggesting good absorption of ammonia by microalgae, as well as its nitrification by bacteria. As a result of the OLR (organic loading rate) increase (Phase 2), a significant (*p* < 0.01) enrichment in the NH_4_^+^ content was observed, corresponding to a decrease in the number of microalgal cells (see Section 2.3) and a visible destabilization of the treatment process. In response, OLR was lowered in Phase 3, leading to process recovery, greater biomass productivity, and improved overall treatment efficiency. No undesirable accumulation of NO_2_^−^ was observed throughout the experiment, and their concentrations did not exceed 0.5 mg/L. The overall TN removal rate (calculated for the steady-state period of 26–33 weeks) was 96.01 ± 0.59%, and was probably related to both microalgal and bacterial activity. The high pH of the photobioreactor environment (pH = 9.89 ± 0.51 calculated for the entire experimental run) (Figure 1a) might have also contributed to the removal of ammonia via stripping. Initially, the pH value increased, most likely due to microalgal uptake of CO_2_ and HCO_3_^−^ [25], as shown in Figure 1. In algae–bacteria systems, CO_2_ produced by bacteria is taken up by algae, which, in turn, produce oxygen utilized by these bacteria, promoting mutualistic performance [26,27,28]. In Phase 2, a pH decline coincided with a statistically significant rise in dissolved CO_2_ (*p* < 0.01), suggesting diminished algal activity under elevated OLR and associated reactor stress. The data imply that the increased OLR predominantly inhibited microalgae, while bacterial populations, typically more tolerant of operational fluctuations, were less affected. The change of operational conditions also affected the removal of organic compounds, indicated by sCOD (Figure 1c). The sCOD value remained stable in Phase 1, then increased significantly in Phase 2 (*p* < 0.01), corresponding to the reduction in microalgal density and suggesting possible cell lysis. When the OLR was reduced to the initial value in Phase 3, sCOD stabilized and remained at a similar level to that reported in Phase 1 (no statistical differences, *p* = 0.493). In the steady state period (26–33 weeks), the calculated sCOD removal rate reached an average value of 88.82 ± 0.60%, with a mean concentration of 716.74 ± 38.27 mgO_2_/L in the reactor effluent. Although some microalgae are able to directly absorb organic carbon [29], the removal of organic substances in mixed systems is mainly performed by bacteria [30,31], thus supporting previous claims. Changes in PO_4_^3−^ concentration throughout the treatment were similar to those of ammonium nitrogen, as illustrated in Figure 1a. Within the first few days of semi-continuous reactor operation, a sharp drop in PO_4_^3−^ was observed (Figure 1a), and the PO_4_^3−^ content remained at a very low level, with an average of 2.60 ± 0.82 mg/L during steady state period, giving an almost 100% removal rate. However, similar to ammonium nitrogen and sCOD, in the 17th week of the experimental run, an increase in PO_4_^3−^ was observed, which could be linked to the microalgae atrophy as mentioned earlier. It is evident that phosphates were utilized by algal biomass and, to some extent, incorporated by bacteria, but due to the high pH, this nutrient might have also been removed via precipitation [32]. The achieved high removal rates of TN, sCOD, and PO_4_^3−^ were noticeably greater than those reported in several previous studies [33,34,35,36,37]. It should be noted that few reports demonstrated even higher treatment efficiencies, although the authors applied different operational conditions, often using dilution, autoclave, or pH adjustment [38,39,40]. In contrast, the methodology applied in this study required no pretreatment, which is commonly used in most published research. A detailed comparison of the results obtained in this study with previous findings is provided in Table S2.

### 2.3. Microalgal Community and Productivity

**Figure 2:**
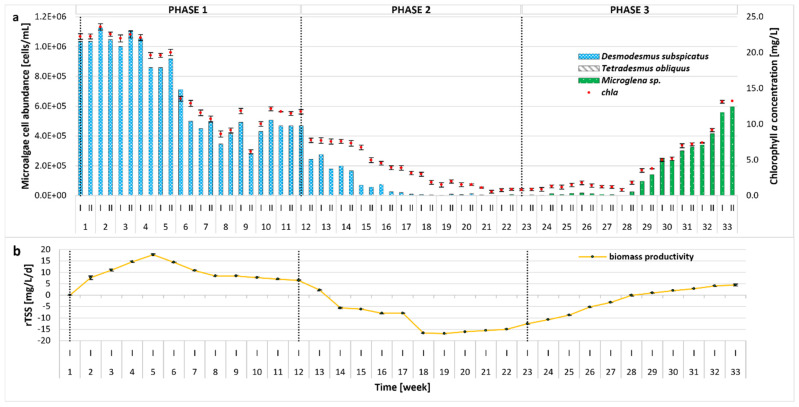
Evolution of biomass concentration and microalgal community distribution throughout the whole 33-week experimental period at different OLRs (0.1, 0.15, and 0.1 g COD/L/d in Phases 1–3, respectively); (**a**) number of microalgal cells [cells/mL] and chla (chlorophyll *a*) concentration [mg/L]; (**b**) rTSS (TSS production rate) [mg/L/d].

Changes in the microalgal community, the growth parameters, and biomass productivity were monitored over the whole experimental period and are illustrated in Figure 2. After the initial batch cultivation period (Phase 0, not included in Figure 2), the microalgal community became almost entirely dominated by *Desmodesmus subspicatus* in the subsequent Phase 1. The remaining strains used to inoculate the bioreactor (*Tetradesmus obliquus*, *Microglena* sp.) were present only in small amounts (4 × 10^2^ cells/mL). Factors affecting such distribution could include light intensity, type of bioreactor (e.g., airlift), or metabolites produced by the microalgal species. In our previous studies, an identical inoculum was successfully used, with a different type of bioreactor (bubble column with thinner glass walls) and lower light intensities of 2200 Lux [18] and 3500 Lux [17] applied; however, the self-shading phenomenon was also observed. To maximize the production of high-value compounds (e.g., lipids), an irradiation of 60–700 µmol/m^2^/s is required [41]; therefore, in the present study, the illuminance was increased to 68.57 µmol/m^2^/s. The results indicate that higher light intensity and the photobioreactor’s type and size (10 L versus 5 L or 350 mL) can be more favorable for the growth of *D. subspicatus.* Additionally, the higher pH of the reactor environment, most likely caused by the photosynthetic activity of *D. subspicatus,* could have inhibited the growth of the other algae species. It is worth noting that the growth of *T. obliquus* is strongly dependent on the pH of the environment, with the optimal value around 7 [42]. On the other hand, the pH preferences of *Microglena* sp. have not yet been established, although it can be concluded that it can grow in an alkaline environment, as observed in Phases 2–3. Even though studies suggest that neutral to alkaline pH may be generally favorable for *Desmodesmus* growth and nutrient removal [43,44], this microalgae is also known for its tolerance to pH fluctuations [45]. Until the 17th week of the experiment, the weekly changes in pH were high. Since *D. subspicatus* is tolerant to such shifts, the growth of *Microglena* was limited, increasing in biomass only when the pH was more stable. Moreover, *D. subspicatus* prefers moderate to high nitrate levels for optimal growth and metabolite production [46]. The decrease in nitrate and orthophosphate concentrations in Phase 3 could have an impact on *D. subspicatus* metabolism, resulting in growth suppression and a shift in species composition. In addition, the initial growth inhibition of *Microglena* and *Tetradesmus* may have resulted from metabolites produced by *D. subspicatus* or competition for nutrients. Microalgae are capable of producing an extraordinary variety of biologically active compounds [47]. These include toxic secondary metabolites such as allelochemicals, which can affect the growth of other organisms. Some species exhibit allelopathic activity towards other microalgae or cyanobacteria, favoring their dominance in the environment [48,49]. As the light intensity remained constant throughout the experiment, no variable light-dependent effects were observed on the microalgal community composition.

During the first 5 weeks of Phase 1, biomass concentration remained relatively stable, with a chla content of 19.60–23.57 mg/L (cell density of 8.59 × 10^5^–1.12 × 10^6^ cells/mL). Productivity showed an overall increasing trend. From week 6, biomass productivity slightly decreased, stabilizing by the end of Phase 1. In response to the higher organic loading rate applied in Phase 2, a sharp reduction in microalgae cell density, chla concentration, and biomass productivity was observed, which correlated with higher concentrations of organic pollutants (COD) and nutrients, as discussed in Section 2.2. In response to suboptimal culture conditions, microalgal growth was disturbed, and the dominance of individual species changed. Under these conditions, the number of *D. subspicatus* gradually decreased, whereas the growth of *Microglena* sp. was observed, further supporting an allelopathic relationship between *D. subspicatus* and *Microglena* sp. as mentioned above. Since the overall microalgae abundance was still very low in Phase 2, the OLR was reduced to the value established in Phase 1. As a consequence, re-acclimatization (Phase 3) and an increase in both the number of microalgae cells and the concentration of chla occurred. A strong correlation between cell count and chla (R > 0.98) and a moderate correlation between OD (optical density at 680 nm) and chla (R > 0.71), OD, and cell count (R > 0.70) were observed. This can be attributed to the fact that OD measurements are influenced not only by the number of cells or pigment content, but also by factors such as cell size, shape, aggregation, and the presence of extracellular substances. These factors can affect the turbidity of the culture, leading to discrepancies between optical density and direct measures of biomass or pigment concentration. In contrast, the strong correlation between cell count and chla reflects the direct relationship between these two parameters in microalgal cultures. Similar observations have been reported in previous studies [50,51]. In Phase 3, an increasing tendency in rTSS was visible. Peak biomass productivity reached nearly 18 mg/L/d, which is comparable to the values reported in the literature for similar microalgae-based systems [34,52]. When stress conditions reduced algal growth, biomass productivity declined, the nutrient removal rate lowered, and the concentrations of ammonium nitrogen, orthophosphates, and sCOD in the effluent increased, resulting in a lowering of the overall process performance as compared to the literature [38].

**Figure 3:**
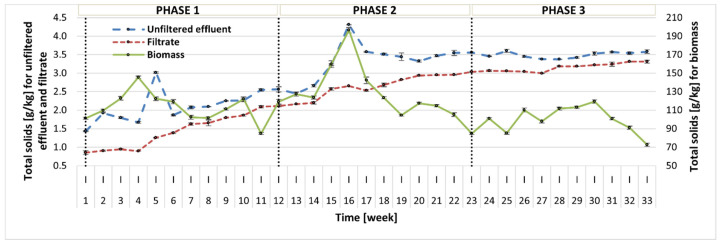
TS content in three fractions: before filtration (unfiltered effluent), after filtration (filtrate), and in the biomass.

In this study, the content of TS and VS was measured in three fractions: (1) photobioreactor effluent before filtration (unfiltered effluent); (2) photobioreactor effluent after filtration (filtrate); (3) biomass (centrifuged photobioreactor effluent). The TS concentration measured in the reactor effluent showed an increasing trend throughout the whole operation, but the TS values in all phases did not differ significantly (2.08–3.50 g/kg) (Figure 3). Simultaneously, the content of VS in TS had a reverse trend and the average VS values reported in the subsequent phases were 56.97 ± 9.61% TS, 47.75 ± 5.91% TS, and 32.61 ± 5.00% TS. This can be linked to changes in the microbial community in the reactor. A similar tendency was reported for both TS and VS contents in the filtrate. It seems that in Phase 2 and especially 3, the liquid fraction contained more soluble inorganic compounds, which could be attributed to debris from the biomass lysis. In contrast, the biomass (centrifuged reactor effluent) displayed an initial increase throughout Phase 1 and Phase 2 until week 16, and during this period, the growth of microalgae was also observed. From that point on, the treatment process broke down, which was reflected by the increase in sCOD, as discussed earlier. In Phase 3, the biomass content stabilized. The average TS concentrations reported in Phases 1–3 were 112.96 ± 16.00 g/kg, 130.34 ± 27.04 g/kg, and 98.86 ± 14.31 g/kg, and the corresponding average VS values were 86.09 ± 4.22% TS, 87.60 ± 3.41% TS, and 81.31 ± 3.74% TS, respectively.

### 2.4. Bacterial Community

**Figure 4:**
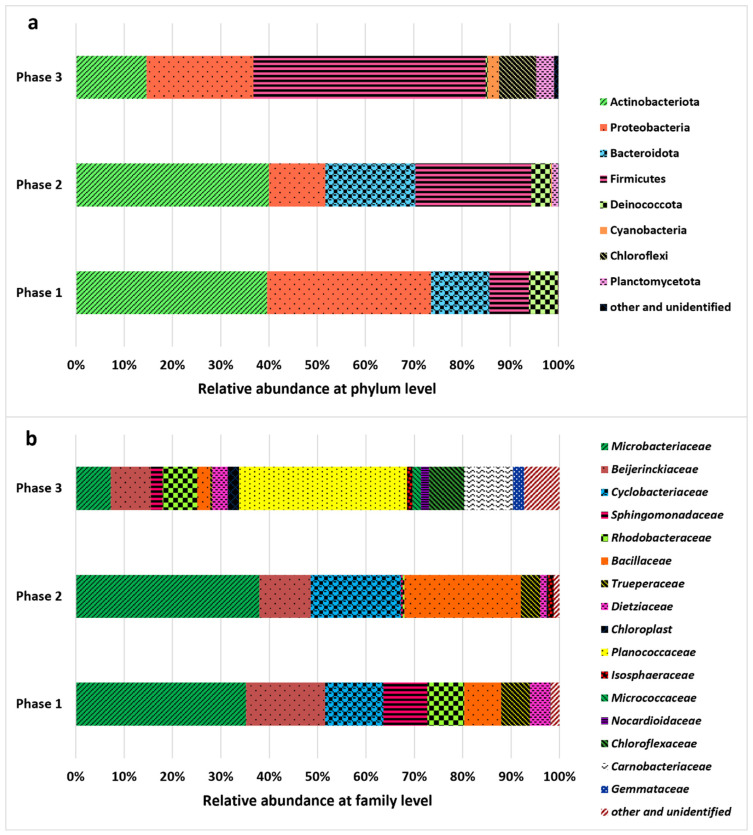
Changes in the relative abundance of bacteria recorded during the operational phases of the experiment (Phase 1: 0.1 g COD/L/day; Phase 2: 0.15 g COD/L/day; Phase 3: 0.1 g COD/L/day); (**a**) phylum level, (**b**) family level.

The structure and diversity of the bacterial population were assessed in the three phases of the reactor operation, and the results are shown in Figure 4. The presence of bacteria resulted from the use of non-axenic cultures and the lack of digestate sterilization. Sterilization was not applied to maintain a more realistic and cost-effective treatment approach. Indigenous bacteria are regarded as highly beneficial in photobioreactor systems because they build microalgae–bacteria consortia responsible for the efficient removal of both nutrients and organic contaminants, which is especially important in large-scale systems [53]. For completely sterile conditions, axenic pure cultures are necessary. However, as previously demonstrated in the literature [54,55], non-axenic cultures can positively affect treatment processes and are more stable. The bacterial community was dominated by two main phyla. Initially, in Phases 1 and 2, the Actinomycetota phylum dominated, constituting approximately 40% of the total community. Then, in Phase 3, Firmicutes dominated the bacterial community, with a relative abundance of 48.15%. The Proteobacteria phylum was also characterized by a relatively high abundance in Phases 1 and 3, accounting for 33.99% and 22.13%, respectively. In Phase 2, Firmicutes constituted 24.08% of relative abundance. Bacteria significantly influenced the transformation of organic compounds and nutrients in the photobioreactor, contributing to their removal [56]. Degradation of organic compounds (expressed by COD removal) can be especially associated with the activity of Firmicutes, Proteobacteria, and Actinomycetota [57], also determined in the photobioreactor. The increase in sCOD reported in Phase 2 may have resulted from the lysis of both microalgae and bacterial cells [7], especially involving Proteobacteria (relative abundance: 33.99% in Phase 1, 11.69% in Phase 2, and 22.13% in Phase 3). Several Proteobacteria species are also involved in the removal of nitrogen and phosphorus from wastewater [58,59]. At the order level, Micrococcales, represented primarily by the *Microbacteriaceae* family, dominated in Phases 1 and 2. Members of this family may play an important role in the denitrification process [60]. High relative abundance of *Beijerinckiaceae* (Rhizobiales) capable of fixing nitrogen [61] was reported in Phase 1, whereas *Bacillaceae* (Bacillales), commonly known for dissimilatory nitrate reduction to ammonium [62,63], were frequent in Phase 2. In turn, Bacillales, primarily the *Planococcaceae* family, were abundant in Phase 3; members of this bacteria are capable of utilizing carbohydrates [64]. Considering the changes in physicochemical indicators discussed in previous sections and the characteristics of the identified microbial community, bacterial activity had a clearly profound effect on digestate treatment.

### 2.5. Elemental Analysis

The samples of the biomass (centrifuged effluent) from each phase were subjected to elemental analysis to evaluate their potential application as fertilizer or in biogas production. The concentrations of macro-elements did not significantly differ between biomass collected in the subsequent phases, and the main component was carbon (67.2–68.2% TS), followed by hydrogen (4.9–5.2% TS). The content of nitrogen varied between 3.25 and 3.75% TS, which is relatively high considering the use of biomass for fertilization. The calculated C/N ratio of approximately 20 makes this biomass attractive for biogas production. The concentration of phosphorus was relatively low (0.24–0.36% TS), whereas sulfur accounted for 0.09–0.11% TS. 

### 2.6. Biochemical Characterization of Biomass

An essential part of the research was to ascertain the valorization potential of biomass generated during digestate treatment. In this study, two types of biomass, BM1 (biomass 1) and BM2 (biomass 2), differing in storage methods (see Section 3.4.5), were evaluated and statistically compared using a *t*-test, as summarized in the Supplementary Materials Section (Table S3).

#### 2.6.1. Biomass Analysis by Attenuated Total Reflectance-Fourier Transform Infrared Spectroscopy

**Figure 5:**
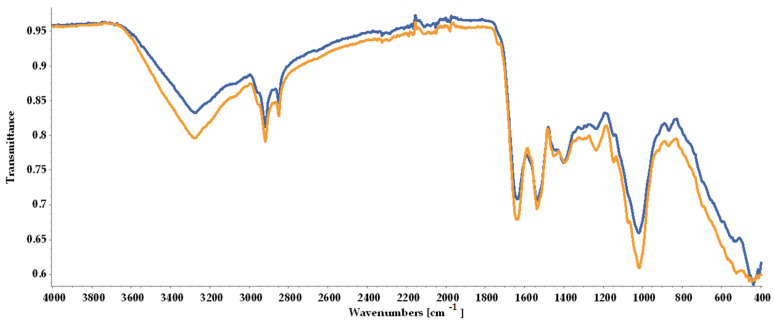
FTIR spectra of dried biomass collected at the end of the experimental process and stored under different conditions prior to analysis: BM1 (yellow line) freeze-dried immediately; BM2 (blue line) stored in tightly closed containers at room temperature for 6 days before freeze-drying.

As shown in Figure 5, the ATR-FTIR (Attenuated Total Reflectance-Fourier Transform Infrared) spectra indicated the loss in biomass quality, with peaks registered for BM2 being less intense than for BM1, although the overall similarity rate was 89.17%.

The ATR-FTIR analysis indicated that the samples possibly contained lipids due to the strong peaks observed at 2851 cm^−1^ and 2919 cm^−1^**,** representing methyl groups (CH_3_ and CH_2_). A higher peak was observed for BM1. Proteins linked to the presence of an amide chemical bound at 1537 cm^−1^ and a protein-linked strong peak at 1637 cm^−1^ were registered. Polysaccharides and carboxylates were noted with one strong peak at 1019 cm^−1^ and a low-intensity peak at 1397 cm^−1^. CH_2_ stretching identified at 1451 cm^−1^ was attributed to carotenoids (Figure 5, Table 2). The obtained results supported the subsequent quantification of lipids, proteins, and pigments in both biomass types.

#### 2.6.2. Lipids

It was observed that the share of individual lipid fractions strongly depends on the type of biomass and, therefore, the method of its storage. BM1 contained, on average, 5.83 ± 0.15 mg of neutral lipids (NLs), 13.33 ± 0.55 mg of glycolipids (GLs), and 4.47 ± 0.29 mg of phospholipids (PLs). For BM2, a comparable amount of GLs was detected (13.87 ± 0.32 mg), but with twice lower content of NLs (3.40 ± 0.26 mg) and 2.5 times higher load of PLs (11.23 ± 0.58 mg). The overall total lipid content was significantly (*p* = 0.025) higher in BM2 (19.00% TS) than in BM1 (15.76% TS). Hence, a 6-day interruption in freezing conditions resulted in concentration changes of PLs and especially NLs, contributing to an overall increase in total lipids. Nonetheless, these values are similar to those reported in Jiang et al. [73] and Tan et al. [74], and slightly lower than those in Feng et al. [75]. The concentration of lipids and their composition determine the further industrial application of the microalgae biomass. NLs, primarily triacylglycerols (TAGs), are the preferred compounds for biodiesel production due to their conversion efficiency and fuel properties [76,77,78]. On the other hand, polar lipids such as PLs and GLs with high unsaturation levels (PUFAs) can negatively affect the conversion process and the final properties of the fuel [76]. The higher content of NLs and the decreased level of PLs in BM1 make this biomass more suitable for biofuel production. In contrast, the greater concentration of PLs and GLs in BM2, combined with the higher total lipid yield, may be beneficial when using biomass in feed, nutraceutical, or cosmetic industries [79,80]. Further research is, however, needed to confirm these findings.

#### 2.6.3. Fatty Acids

FAs (fatty acids) constitute the majority of lipid molecules and can be the components of both polar and neutral lipids [81]. It is known that microalgae are able to synthesize a variety of fatty acids, including saturated (SFA), monounsaturated (MUFA), and polyunsaturated (PUFA) [82], all of which were found in both biomass types. For NLs, GLs, and PLs, respectively, 11, 7, and 6 different SFAs; 6, 4, and 4 MUFAs; and 5, 3, and 2 PUFAs were detected in BM1. Likewise, in BM2, 2, 5, and 2 SFAs; 2, 2, and 3 MUFAs; and 2, 2, and 1 PUFAs (Table 3) were found for NLs, GLs, and PLs, respectively.

The main FAs detected in both biomass types were saturated palmitic (C16:0) and stearic (C18:0) acids. For BM1, the highest palmitic acid content was determined, accounting for 28.17% (NLs), 40.79% (GLs), and 53.68% (PLs) of total lipids. In BM2, C16:0 accounted for 44.38%, 43.75%, and 55.13% of the corresponding fractions of total lipids, respectively. A high stearic acid content in BM1 was recorded, with 18.51% (NLs), 26.65% (GLs), and 16.85% (PLs) of total FAs. In turn, BM2 C18:0 constituted 30.97%, 30.16%, and 20.76% of all FAs in the respective fractions. The reported values can be linked to the long culture cycle of microalgae, which induces cells to accumulate reserve lipids rich in SFA (C18) used for carbon storage [83]. Feedstocks rich in MUFA are desirable in biodiesel production, but the composition of SFA is also important [84]. In most substrates used to produce biodiesel from vegetable oils, major fatty acids are oleic, linoleic, palmitic, and stearic [85]. It is worth noting that these constituted the majority of total FAs found in both biomass types. Additionally, MUFA accounted for a higher percentage of total FAs than PUFA. These results suggest that both biomass types could potentially be used for biodiesel production. Both were also characterized by a high content of gondoic acid, the presence of which has also been observed in the crop plant *Camelina sativa,* which is suitable for the production of aviation fuel, biodiesel, and industrial grease [86].

#### 2.6.4. Proteins and Pigments

Microalgae are a promising source of proteins and may provide a sustainable solution to the growing world population and food demand [15]. For many microalgal species, protein content varies between 6 and 63% TS, with most species containing 40% TS. The scientific literature indicates that the efficiency of protein production strongly depends on the strain type, growth phase, and is significantly influenced by the environment in which the microalgae grow [87]. The protein content reported in this study was between 32.75% TS (BM1) and 33.59% TS (BM2). The obtained values are similar to those present in *Euglena gracilis* (Euglenophyta), *Porphyridium purpureum* (formerly *Porphyridium cruentum*) (Rhodophyta), *Galdieria sulphuraria* (Rhodophyta), and conventional sources such as milk and soy [88]. A relatively high concentration of proteins makes the microalgal biomass attractive for feed production [15].

The main pigments detected in both biomass types were chlorophylls and lutein (Table 4). Specifically, BM1 was most abundant in neoxanthin and β-carotene, while BM2 contained almost 10% more chla; however, a *t*-test analysis showed no statistical differences. Other pigments, such as violaxanthin, lutein, and chlb (chlorophyll *b*), were present at comparable levels. The concentration of total chlorophyll (chla + chlb) was greater in BM2, whereas carotenoids displayed higher amounts in BM1. In both cases, some statistical differences were reported (*p*-value of 0.032 and 0.029, respectively). Therefore, the storage method influenced both the pigment type and concentration, similarly to the findings reported by Supakorn et al. [89]. This phenomenon was also confirmed by Gouveia et al. [90], who observed an increase in the concentration of carotenoids in microalgal biomass after 1 week of storage in freezing conditions compared to samples kept at room temperature. Chlorophylls from microalgae could potentially be used as food ingredients or food dyes. In addition, they demonstrate high bioactivity, have anti-cancer and antioxidant properties, and may improve the immune system [91]. Likewise, chlorophylls and lutein can be used as food coloring or in functional foods. Lutein also has antioxidant, anti-atherosclerotic, and anti-inflammatory properties, and can strengthen immunity. It is also used as a feed additive or to brighten the colors of poultry feathers and deepen the yellow shade of egg yolks [91,92].

## 3. Materials and Methods

### 3.1. Liquid Substrate

Digestate was collected from the anaerobic digestion tank at the municipal wastewater treatment plant in Lodz, Poland (51°43′34.434″ N 19°20′50.697″ E). The liquid fraction was obtained by mechanical dewatering (belt press) of the anaerobic sludge conducted at the treatment plant, with coagulants and organic flocculants used to improve the process. The liquid digestate was not subjected to any pre-treatment methods, including filtration, sterilization, dilution, or pH adjustment prior to the experiments. Throughout the experiment, the digestate was stored in closed plastic containers at 4 °C. Prior to each feeding cycle, the substrate was allowed to reach room temperature. Stability of the raw liquid digestate was analyzed with physicochemical measurements performed in accordance with the methodology presented in Section 3.4.1. Analyses were performed on non-treated substrate before the experimental run and after 11, 22, and 33 weeks. The composition of the liquid digestate is summarized in Table 1. 

### 3.2. Microalgae Inoculum

For the purpose of the study, a non-axenic mixed algal culture of *Tetradesmus obliquus*, *Desmodesmus subspicatus*, and *Microglena* sp. (Chlorophyta) (environmental isolates) was used. Each strain was identified in the previous study described by Sobolewska et al. [93] and deposited in NCBI (National Center for Biotechnology Information; www.ncbi.nlm.nih.gov, accessed on 20 April 2022) under accession numbers ON457158–ON457160 (for the rbcl gene) and ON426490–ON42692 (for the 18S-ITS region). The photobioreactor was inoculated with an algal mixture containing 1.0 × 10^4^ cells/mL of each species in the reactor working volume. 

### 3.3. Photobioreactor Setup and Experimental Organization

A semi-continuous experiment for liquid digestate treatment was carried out in a large-scale photobioreactor (capacity of 20 dm^3^, working volume of 10 dm^3^) with continuous aeration (flow rate of 0.7 L/L/min, as in the previous studies [18,93,94]) using a membrane blower (Yasunaga, Japan). Light-Emitting Diode (LED) lamps (color temperature = 6000 K; luminous power = 1440 lm; electric power = 14.5 W) were used to illuminate the bioreactor in a photoperiod of 14 h light and 10 h dark. The illumination intensity was set at 4800 Lux (68.57 µmol/m^2^/s). Compared to previous experiments, increased light intensity was applied due to a self-shading phenomenon typically observed during long-lasting treatment processes [94,95,96]. Furthermore, it was documented that greater light intensity of 60–700 µmol/m^2^/s promotes the formation of high-value metabolites, including lipids [41]. To allow for multiplication of algal biomass, the bioreactor was filled with 3N-BBM (Bold’s Basal Medium with 3-fold nitrogen concentration) and distilled water in a volume ratio of 1:9, and inoculated with 10% *v*/*v* algal consortium as described in Section 3.2. The 3N-BBM was prepared following the protocol described in Andersen [42]. The reactor was initially operated in batch mode for 4 weeks (algae multiplication; hereinafter referred to as Phase 0), and then a semi-continuous treatment process was started, lasting up to 33 weeks. During the semi-continuous operation, feeding and discharge of the photobioreactor were performed using a peristaltic pump. Two OLR values were applied to evaluate reactor performance and biomass growth under variable operating conditions. The OLR value was initially set at 0.1 gCOD/L/day (Phase 1), corresponding to hydraulic retention time of 30 days, adopted from previous experiments. In Phase 2, the OLR value was increased to 0.15 gCOD/L/day, and finally reduced to 0.1 gCOD/L/day (Phase 3) as a result of process instability. Each operational phase was continued for 11 weeks.

Twice a week, pH, concentration of nitrogen compounds (i.e., ammonium nitrogen, nitrates, nitrites), and soluble phosphates were measured after filtration. CO_2_ was determined once a week in non-filtered samples, while sCOD was measured once a week in filtered samples. The analyses of turbidity, TSS, chla concentration, cell number of microalgae, and OD were measured in unfiltered samples twice a week. Additionally, once a week, TS and VS were measured in effluent, filtrate, and biomass. Treatment efficiency was calculated based on nitrogen, phosphates, and sCOD removal for the steady-state period (26–33 weeks), using raw liquid digestate as a control sample. Steady-state operation was determined as the experimental period throughout which most of the measured indicators did not change by more than 10%. Biomass was collected from the photobioreactor twice a week, then frozen and stored in closed plastic containers at −20 °C. At the end of the operation, the algal biomass was subjected to a series of tests that included elemental analysis, ATR-FTIR (Bruker, Ettlingen, Germany), total and lipid fractionations, fatty acid evaluation, and extraction and quantification of proteins and pigments.

### 3.4. Analytical Methods

#### 3.4.1. Physicochemical Tests

TS, VS, and pH were analyzed in accordance with Standard Methods [97]. Physicochemical indicators were determined using a UV–VIS DR6000 spectrophotometer (Hach Lange, Loveland, CO, USA) and Hach Lange kits following the manufacturer’s procedures. TN was determined as the sum of nitrogen present in ammonium nitrogen, nitrates, and nitrites, which were measured using the modified Nessler (no. 8038), NitraVer 5 (no. 8039), and NitriVer 3 (no. 8507) tests. Orthophosphates, total volatile fatty acids, COD, and CO_2_ were measured using PhosVer 3 (no. 8048), LCK365, LCK214, and LCK388 tests, respectively. Additionally, a thiocyanate method no. 8113 (for chlorides), a SulfaVer 4 method no. 8051 (for sulfates), and a methylene blue method no. 8131 (for sulfides) were applied. The concentrations of zinc, iron, copper, and aluminium were determined by Zincon method no. 8009, FerroVer method no. 8008, CuVer 1 method no. 8506, and AluVer method no. 8012, respectively. In addition, EN ISO 7027 (for turbidity) and photometric method no. 8006 (for TSS) were used. Color was measured using the Platinum–Cobalt standard method (no. 8025), with division into apparent color (determined in unfiltered samples) and true color (determined in filtered samples).

#### 3.4.2. Microalgae Biomass Parameters

Throughout the reactor operation, algal growth parameters were monitored by determining microalgae cell abundance, chla concentration, and OD. Tests were performed according to the methodology described in the previous study [18]. Biomass productivity was assessed based on the TSS indicator and calculated in accordance with the methodology described in Min et al. [98].

#### 3.4.3. Metagenomic Analysis

To determine the biodiversity of the bacterial community, a metagenomic analysis was performed on non-pooled biomass samples collected during the last week of each experimental phase. This approach allowed for the assessment of changes in microbial community structure in response to the conditions prevailing in each phase of the process. The analysis was carried out based on the hypervariable V3–V4 region of the 16S rRNA gene, according to the methodology described in Sobolewska et al. [17]. The metagenomic data have been deposited into the NCBI database. The Sequence Read Archive tool was used to create a bio-project with accession number PRJNA1185632 (BioSampleAcc. SAMN44716321, SAMN44716322, SAMN44716323). The obtained fragments of the 16S rRNA gene were matched against the appropriate taxonomical levels (from phylum to species) to determine phylogenetic microbial diversity.

#### 3.4.4. Elemental Analysis

Trace element composition (C, N, H, P, S) of the biomass was determined using a Flash Elemental Analyzer (Thermo Finnigan, Italy) in accordance with the manufacturer’s procedures. Based on this analysis, the C/N ratio was then calculated.

#### 3.4.5. Algae Biomass Valorization

The biomass collected from the photobioreactor was thoroughly mixed and divided into two parts, hereinafter referred to as BM1 and BM2. Both were used for the determination of value-added compounds. Before measurements, samples were freeze-dried using Scanvac CoolSafe (Labogene, Denmark). Prior to freezing, BM2 was additionally stored under limited oxygen conditions for 6 days in airtight containers at room temperature, allowing for its partial decomposition. This procedure simulated short-term storage that may occur during laboratory or pilot-scale operations, where immediate processing is not always feasible.

##### ATR-FTIR Spectroscopy

ATR-FTIR was employed to characterize the structure of dried microalgae biomass. The infrared (IR) spectra (24 scans) were obtained at room temperature (referenced against air) in the wave number range of 400–4000 cm^−1^ (resolution of 4 cm^−1^) using a Bruker Alpha II (Bruker, Ettlingen, Germany). Spectra were analysed with OPUS 7.2 software (Bruker, Ettlingen, Germany). For the exploratory analysis, a multi-linear regression (singular value decomposition) was used to estimate the relationship between each sample using the Spectragryph program (Spectragryph, Oberstdorf, Germany) [99] in the spectral range of 400–4000 cm^−1^.

##### Total Lipids Determination

Extraction was performed based on the methodology described by Matyash et al. [100]. A total of 6 mL of methanol was added to 150 mg of dry biomass, vortexed, and transferred to 250 mL flasks. A total of 20 mL of methyl-tert-butyl ether (MTBE) was then added and incubated with agitation (100 rpm, 1 h), followed by the addition of 5 mL ultrapure water and incubation at room temperature for 10 min. The contents were centrifuged (4500 rpm, 10 min) using a Heraeus Megafuge 8 (Thermo Scientific, Hanau, Germany). The upper organic phase was transferred to evaporation flasks, and the lower phase was returned and MTBE/methanol/ultrapure water was added in a volume ratio of 20:6:5. Incubation with agitation (100 rpm, 1 h) was repeated, and the upper organic phase after centrifugation (4500 rpm, 10 min) was transferred to evaporation flasks. Suspension of the lower phase in MTBE/methanol/ultrapure water (20:6:5 *v*/*v*/*v*), incubation with agitation, centrifugation, and transfer of the upper organic phase were repeated until complete lipid extraction was achieved. Extracts were completely dried using a Rotavapor R-300 (Büchi, Barcelona, Spain), then resuspended in chloroform and dried using a speed-vacuum centrifuge (Gyrozen, Seoul, Republic of Korea).

##### Separation of Lipid Fractions

Lipids extracted in Section Total Lipids Determination were suspended in chloroform, vortexed, and sonicated until completely dissolved. Acquired samples were transferred to the top of a silica gel column. For elution of neutral lipids, glycolipids, and phospholipids, chloroform, acetone, and methanol were added, respectively. Each fraction was collected in separate tubes, transferred to individual evaporation flasks, and dried using a Rotavapor R-300 (Büchi, Spain). Dried samples were resuspended in the appropriate solvent (chloroform/acetone/methanol) and centrifuged (12,000 rpm, 15 min) using a SIGMA 1–14 microcentrifuge (SIGMA, Darmstadt, Germany). Samples were dried using a speed-vacuum centrifuge (Gyrozen, Republic of Korea) [101].

##### Fatty Acid Analysis

For each lipid fraction (i.e., neutral lipids, glycolipids, and phospholipids), chromatographic analysis of fatty acids was performed. For this purpose, 1 mL of hexane and 500 µL of methanol were introduced to each fraction, vortexed, and/or sonicated until dissolution. Then, 400 µL sodium methoxide was added to the prepared samples, vortexed for 5 min, and the upper phase was filtered using a nylon syringe filter (ϕ = 0.22 µm). The extract was transferred to new vials, and an internal standard—methyl nonadecanoate (C19:0) (Sigma-Aldrich, Algés, Portugal)—was introduced at a concentration of 0.3 mg/mL. Chromatographic analysis of fatty acids was carried out using a gas chromatography apparatus NEXIS GC2030 (Shimadzu, Japan) equipped with a flame ionization detector (FID) and a TR-CN 100 capillary column (60 m × 0.25 m × 0.20 µm). As the carrier gas (at a pressure of 150 kPa), helium was used. The injector and detector temperatures were set at 260 °C, and the split ratio corresponded to 1:25. The initial column temperature was kept at 90 °C for 7 min after the injection. The temperature increased by 5 °C per minute until 220 °C was reached and held for more than 15 min. Data were collected and analyzed by Lab Solutions analysis software. Fatty acids were identified by comparison of the relative retention times with an authentic external standard—Supelco 37 component FAME mix. Quantification was performed using the internal standard method [102]. Results were expressed as a percentage of total FAs.

##### Protein Extraction and Quantification

Protein extraction and quantification were performed based on the methodology adopted from Lowry et al. [103] and Barbarino and Lourenço [104]. Glass beads and 1 mL of cold ultrapure water were added to 12.5 mg of dry biomass. The samples were homogenized (30 cycles/s, 10 min) using a bead mill (Retsch, Haan, Germany), then 1 mL of ultrapure water was added, and the samples were centrifuged (4500 rpm, 10 min) using a Heraeus Megafuge 8 (Thermo Scientific, Germany). The supernatant was collected and stored on ice. Then, 4 mL of 0.1 N NaOH was added to the pellet, agitated for 30 min at room temperature, and the samples were centrifuged (4500 rpm, 10 min). The supernatant was collected, and the addition of NaOH, agitation, and centrifugation were repeated until complete protein extraction. In order to quantify proteins, a calibration curve was prepared based on Bovine Serum Albumin (BSA). A volume of 200 µL of BSA (0.3 mg/mL; 0.25 mg/mL; 0.2 mg/mL; 0.15 mg/mL; 0.1 mg/mL; 0.05 mg/mL; 0.025 mg/mL; 0.01 mg/mL) or test samples were mixed with 1 mL of complex reagent (2 g NaOH + 10 g Na_2_Co_3_ + 500 mL H_2_O (Reagent A) + 0.1 g KC_4_H_4_NaO_6_ × 4H_2_O + 50 mg CuSO_4_ × 5H_2_O + 10 mL H_2_O (Reagent B)) in a 50:1 v/v ratio. The samples were vortexed and then incubated for 20 min in the dark at room temperature. Then, 100 µL of 1N Folin Ciocalteu reagent was added to the samples, vortexed, and incubated for 35 min in the dark at room temperature. Absorbance was read at 750 nm using a Jenway 6850 UV/Vis Spectrophotometer (Jenway, UK).

##### Total Carotenoids and Total Chlorophylls Determination

For the determination of total carotenoids and total chlorophylls, the procedure described in Stengel and Connan [105], Lichtenthaler [106], and Bauer and Minceva [107] was applied. Liquid nitrogen was added to 50 mg of dry biomass, and after complete evaporation, glass beads and 1 mL of 100% methanol were introduced. Samples were homogenized (30 cycles/s, 15 min) using a bead mill (Retsch, Germany), followed by the addition of 4 mL of 100% methanol and centrifugation (2000 rpm, 10 min). The 20-fold diluted supernatant was used to perform a spectrum scan between 400 nm and 700 nm, and then to measure the concentrations of chlorophyll *a*, chlorophyll *b*, total chlorophyll *a* and *b*, and carotenoids according to Equations (1)–(4). Pure methanol was used as a blank sample.

chla (μg/mL) = (16.72 × A665.2 − 9.46 × A652.4) × 20(1)

chlb (μg/mL) = (34.09 × A652.4 − 15.28 × A665.2) × 20(2)

chla + b (μg/mL) = (1.44 × A665.2 + 24.93 × A652.4) × 20(3)

car (μg/mL) = ((1000 × A470 − 1.63 × Chla − 104.96 × Chlb)/221) × 20(4)

where chla—chlorophyll *a* concentration; chlb—chlorophyll *b* concentration; chla + b—total chlorophyll *a* and *b* concentration; car—carotenoids concentration; A665.2—absorbance at the wavelength of 665.2 nm; A652.4—absorbance at the wavelength of 652.4 nm; A470—absorbance at the wavelength of 470 nm; 20—dilution factor.

##### Pigments Extraction and Quantification

The methodology for pigment (neoxanthin, violaxanthin, lutein, chlorophyll *a*, chlorophyll *b*, β-carotene) extraction and analysis was adopted from Bauer and Minceva [107], Mendes et al. [108], and van Heukelem and Thomas [109]. Liquid nitrogen was added to 5 mg of dry biomass, and after evaporation, glass beads and 1 mL of MTBE were introduced. Samples were homogenized (30 cycles/s, 15 min) using a bead mill (Retsch, Germany) and then centrifuged (12,000 rpm, 10 min) using a SIGMA 1–14 microcentrifuge (SIGMA, Germany). The extracts were filtered into vials using a PTFE filter (ϕ = 0.45 µm). Samples were examined using an HPLC Nexera XR (Shimadzu, Kyoto, Japan), consisting of a solvent delivery module LC-2AD XR with system controller CBM-20A, photodiode array SPD-M20A, and column oven CTO-20AC. A monomeric Telos C18 column (150 mm × 4.6 mm) was used. The HPLC (high-performance liquid chromatography) procedure included a flow rate = 1.1. mL/min, an injection column = 100 µL, a column temperature = 60 °C, and a run duration = 35 min. The gradient profile and mobile phase (methanol:water; acetonitrile:water; ethyl acetate) composition was compliant with the method described by Mendes et al. [108].

### 3.5. Statistical Analysis

Most measurements were performed in triplicate. Mean values, standard deviations (SD), error bars, and Pearson correlation coefficients (where 0.0–0.2 = very weak correlation; 0.2–0.4 = weak correlation; 0.4–0.7 = moderate correlation; 0.7–0.9 = strong correlation; 0.9–1.0 = very strong correlation) were calculated in Microsoft Excel 365. Statistical comparisons were performed using one-way analysis of variance (ANOVA) and Student’s *t*-test, with a significance level of 0.05.

## 4. Conclusions

The study demonstrated that microalgae–bacteria consortia can be successfully used for the long-term treatment of undiluted, unsterilized, and unfiltered liquid digestate, with the simultaneous synthesis of valuable compounds. A high removal rate of organic contaminants and nutrients—up to 89–99%—was achieved. *Desmodesmus subspicatus* showed greater tolerance to process conditions and exhibited rapid initial growth, while *Microglena* sp. and *Tetradesmus obliquus* were inhibited. An increase in the organic loading rate resulted in destabilization of the microbial community—more visible for microalgae—which led to a reduction in treatment efficiency. The allelopathic activity of *D. subspicatus*, negatively affecting the other species, was identified, while *Microglena* sp. showed higher readaptation and recolonization potential. Furthermore, *Microglena* sp. played a crucial role in achieving high treatment efficiency in the final experimental stage. The impact of the storage regime on the composition of the valorized biomass was also evaluated. Lyophilization and subsequent frozen storage had a positive effect on the content of neutral lipids, which are valuable for biofuel production.

Further research is needed to fully exploit the potential of microalgae in liquid digestate treatment and the production of value-added materials. Since algae consume a lot of light energy for metabolic processes, it is necessary to optimize and improve lighting sources and photobioreactor constructions to lower operational costs. It was demonstrated that bacteria play an important role in photobioreactor installations for liquid digestate treatment. Therefore, a better understanding of the relationship between bacteria and algae is essential. For this purpose, high-resolution molecular and metabolic techniques should be employed. Finally, it is recommended to establish the cost-saving benefits of different storage strategies over varying timeframes and interruption periods.

## Figures and Tables

**Table 1:** Characteristics of raw liquid digestate *.

Indicator	Unit	Average Value ± SD
pH	-	6.50 ± 0.03
Total solids, TS	g/kg	2.88 ± 0.04
Volatile solids, VS	g/kg	1.34 ± 0.03
Volatile solids, VS	% TS	46.54 ± 1.67
Total suspended solids, TSS	mg/L	7.08 ± 0.67
Turbidity	FAU	108.42 ± 2.47
Apparent color	mg/L Pt-Co	409.67 ± 4.10
True color	mg/L Pt-Co	368.92 ± 3.15
Total chemical oxygen demand, tCOD	mgO_2_/L	7414.17 ± 39.65
Soluble chemical oxygen demand, sCOD	mgO_2_/L	6411.67 ± 62.50
Total volatile fatty acids, tVFAs	mg/L	3100.00 ± 76.99
Ammonium nitrogen, NH_4_^+^	mg/L	121.67 ± 6.56
Nitrates, NO_3_^−^	mg/L	1061.67 ± 43.66
Nitrites, NO_2_^−^	mg/L	10.58 ± 1.09
Total nitrogen, TN **	mgN/L	337.58 ± 10.51
Orthophosphates, PO_4_^3−^	mg/L	491.67 ± 26.23
Sulfates, SO_4_^2−^	mg/L	0.00 ± 0.00
Sulfides, S^2−^	μg/L	0.00 ± 0.00
Chlorides, Cl^−^	mg/L	282.50 ± 7.54
Iron, Fe	mg/L	9.34 ± 0.16
Copper, Cu	mg/L	7.06 ± 0.16
Zinc, Zn	mg/L	14.08 ± 0.79
Aluminum, Al^3+^	mg/L	0.64 ± 0.05

* Mean values ± SD from 12 measurements ** The nitrogen sum in all determined nitrogen forms (ammonium nitrogen, nitrates, nitrites).

**Table 2:** ATR-FTIR band identification and characterization of the dried raw microalgae biomass (nd—non-detected; sh—shoulder).

Wavenumber (cm^−1^)	Chemical Bound	BM1	BM2	References
436–445	Aryl disulfides (S-S stretch)	++++	++++	[65]
524–536	Benzene group (C–Br stretch)	++++	+++	[66]
867–869	Bending of the C-H, uronic acid	+	+	[67]
1019–1021	v(C-O-C) of polysaccharides	+++++	+++++	[68]
1237–1238	Phosphate-containing compounds (P = O) stretching of phosphodiesters	+	+	[69]
1307	Deformation vibrations of CH_3_ groups	+	nd	[70]
1397–1402	Carboxylic acid vs(C-O) of COO- groups of carboxylates	++	++	[69]
1451	Scissoring vibrations of CH_2_ groups	++	sh	[70]
1537	Protein amide II band mainly δ(N-H) bending and v(C-N) stretching	+++	+++	[71]
1637–1638	Amide (C-N) and carbonyl (C=O) in protein	+++	+++	[69]
2851	CH_3_-methyl	+++	+++	[71]
2919	CH_2_-methyl	+++	+++	[71]
3276	O-H bounds	++++	+++	[72]

**Table 3:** FA composition of BM1 and BM2 as percentage of total fatty acids (%) (n.d.—not detected).

Individual FAs	Formula	BM1 [% of Total FAs]			BM2 [% of Total FAs]		
		NLs	GLs	PLs	NLs	GLs	PLs
**SFA**							
Capric acid	(C10:0)	2.17 ± 0.15	n.d.	n.d.	n.d.	n.d.	n.d.
Undecylic acid	(C11:0)	1.47 ± 0.24	3.99 ± 0.88	2.20 ± 0.37	n.d.	8.83 ± 0.82	n.d.
Tridecylic acid	(C13:0)	0.51 ± 0.27	1.66 ± 0.18	0.91 ± 0.07	n.d.	3.47 ± 0.32	n.d.
Myristic acid	(C14:0)	n.d.	1.27 ± 0.11	1.00 ± 0.04	n.d.	n.d.	n.d.
Pentadecylic acid	(C15:0)	1.09 ± 0.47	0.94 ± 0.16	n.d.	n.d.	n.d.	n.d.
Palmitic acid	(C16:0)	28.17 ± 1.35	40.79 ± 0.91	53.68 ± 0.56	44.38 ± 0.78	43.75 ± 1.04	55.13 ± 0.69
Margaric acid	(C17:0)	1.06 ± 0.60	n.d.	1.65 ± 0.16	n.d.	n.d.	n.d.
Stearic acid	(C18:0)	18.51 ± 0.88	26.65 ± 1.14	16.85 ± 1.39	30.97 ± 0.58	30.16 ± 1.66	20.76 ± 0.49
Arachidic acid	(C20:0)	3.23 ± 0.16	n.d.	n.d.	n.d.	n.d.	n.d.
Heneicosylic acid	(C21:0)	0.53 ± 0.32	n.d.	n.d.	n.d.	n.d.	n.d.
Behenic acid	(C22:0)	1.91 ± 0.08	n.d.	n.d.	n.d.	n.d.	n.d.
Lignoceric acid	(C24:0)	1.37 ± 0.09	n.d.	n.d.	n.d.	n.d.	n.d.
**Total**		60.02 ± 4.61	75.30 ± 3.38	76.30 ± 2.59	75.35 ± 1.36	86.21 ± 3.84	75.89 ± 1.18
**MUFA**							
Myristoleic acid	(C14:1)	0.80 ± 0.18	n.d.	n.d.	n.d.	n.d.	n.d.
Palmitoleic acid	(C16:1)	1.77 ± 0.08	2.18 ± 0.23	3.12 ± 0.28	n.d.	n.d.	4.70 ± 0.10
Heptadecanoic acid	(C17:1)	1.55 ± 0.13	1.58 ± 0.26	1.29 ± 0.13	n.d.	n.d.	n.d.
Elaidic acid	(C18:1w9 trans)	1.37 ± 0.72	n.d.	n.d.	n.d.	n.d.	n.d.
Oleic acid	(18:1w9 cis)	8.93 ± 0.65	6.09 ± 0.48	6.23 ± 0.35	9.06 ± 2.38	3.85 ± 0.78	7.82 ± 0.06
Gondoic acid	(C20:1w9)	11.22 ± 0.89	7.23 ± 0.88	7.58 ± 0.91	6.52 ± 0.60	5.73 ± 0.45	7.87 ± 0.56
**Total**		25.64 ± 2.65	17.08 ± 1.85	18.22 ± 1.67	15.58 ± 2.98	9.58 ± 1.23	20.39 ± 0.72
**PUFA**							
Linolelaidic acid	(C18:2w6 trans)	2.33 ± 0.14	n.d.	n.d.	n.d.	n.d.	n.d.
Linoleic acid	(C18:2w6 cis)	9.02 ± 0.64	5.37 ± 0.55	4.54 ± 0.30	4.64 ± 0.63	2.32 ± 0.15	3.73 ± 0.32
Gamma-linolenic acid	(C18:3w6)	1.44 ± 0.27	n.d.	n.d.	n.d.	n.d.	n.d.
Eicosadienoic acid	(C20:2)	1.23 ± 0.08	1.14 ± 0.15	0.94 ± 0.23	n.d.	n.d.	n.d.
Eicosatrienoic acid	(C20:3w3)	1.09 ± 0.12	n.d.	n.d.	4.43 ± 1.82	n.d.	n.d.
**Total**		15.11 ± 1.25	6.51 ± 0.70	5.48 ± 0.53	9.07 ± 2.45	2.32 ± 0.15	3.73 ± 0.32
**SFA + PUFA**							
Tricosylic acid + Arachidonate acid	(C23:0) + (C20:4w6)	n.d.	1.11 ± 0.32	n.d.	n.d.	1.89 ± 0.31	n.d.
**Total**		n.d.	1.11 ± 0.32	n.d.	n.d.	1.89 ± 0.31	n.d.

**Table 4:** Biomass pigment content.

Pigment	Unit	Mean Value ± Standard Deviation	
		BM1	BM2
Neoxanthin	%total pigments	5.10 ± 0.82	3.99 ± 0.31
Violaxanthin		10.08 ± 1.62	9.69 ± 0.51
Lutein		21.73 ± 1.65	19.81 ± 0.73
chla		30.52 ± 2.07	39.48 ± 0.12
chlb		21.71 ± 1.47	22.41 ± 0.83
β-carotene		10.88 ± 1.76	4.63 ± 0.18
chla	μg/mL	129.74 ± 6.83	120.30 ± 3.13
chlb		46.66 ± 11.72	86.66 ± 12.25
chla + chlb		178.34 ± 6.71	209.28 ± 9.25
Carotenoids		52.66 ± 6.33	36.38 ± 6.29

## Data Availability

The sequences obtained during the current research were submitted to the National Center for Biotechnology Information under accession number PRJNA1185632 (BioSampleAcc. SAMN44716321, SAMN44716322, SAMN44716323).

## Supplementary Materials

The following supporting information can be downloaded at: https://www.mdpi.com/article/10.3390/molecules30132778/s1, Table S1. Statistical comparison of the experimental phases (*p*-value matrix of the ANOVA test); Table S2. Comparison of experimental procedure, treatment efficiency, and biomass valorization directions; Table S3. Statistical comparison of the BM1 and BM2 biomass (*p*-values of the *t*-test).
