# Supplementary material for: Simultaneous Liquid Digestate Treatment and High-Value Microalgal Biomass Production: Influence of Post-Harvest Storage on Biochemical Profiles
Source: Molecules. 2025 Jun 27;30(13):2778. doi: 10.3390/molecules30132778 (PMC12251123; doi:10.3390/molecules30132778)
Supplement: Supplementary file 1 [file molecules-30-02778-s001.zip › molecules-3672009-supplementary.pdf]

<u>Supplementary material</u>

Microalgae-bacteria liquid digestate treatment with simultaneous generation of high-value biomass

Ewelina Sobolewska[a,b,*], Michał Komar[a,b], Sebastian Borowski[a], Paulina Nowicka-Krawczyk[c], António Portugal[d,e,f], Nuno Mesquita[d], Mariana F. G. Assunção[g], Berk Aksoy[g], João Cotas[h], Leonel Pereira[e,h]

[a]Department of Environmental Biotechnology, Faculty of Biotechnology and Food Sciences, Lodz University of Technology, Wólczańska 171/173, 90-530 Lodz, Poland

[b]Interdisciplinary Doctoral School, Lodz University of Technology, Żeromskiego 116, 90-924 Lodz, Poland

[c]Department of Algology and Mycology, Faculty of Biology and Environmental Protection, University of Lodz, Banacha 12/16, 90-237 Lodz, Poland

[d]Centre for Functional Ecology (CFE)—Science for People & the Planet, Department of Life Sciences, University of Coimbra, Calçada Martim de Freitas, 3000-456 Coimbra, Portugal

[e]TERRA—Associate Laboratory for Sustainable Land Use and Ecosystem Services, Department of Life Sciences, University of Coimbra, Calçada Martim de Freitas, 3000–456 Coimbra, Portugal

[f]FitoLab—Laboratory for Phytopathology, Instituto Pedro Nunes, Rua Pedro Nunes, 3030–199 Coimbra, Portugal

[g]Coimbra Collection of Algae (ACOI), Department of Life Sciences, University of Coimbra, 3000-456 Coimbra, Portugal

[h]Marine Resources, Conservation and Technology, Marine Algae Lab, CFE—Centre for Functional Ecology: Science for People & Planet, Department of Life Sciences, University of Coimbra, 3000-456 Coimbra, Portugal

*Corresponding author. E-mail address: ewelina.sobolewska@dokt.p.lodz.pl

Co-authors' email addresses: michal.komar@dokt.p.lodz.pl; sebastian.borowski@p.lodz.pl; paulina.nowicka@biol.uni.lodz.pl; aportuga@bot.uc.pt; inunomesquita@gmail.com; mariana.assuncao@uc.pt; berk.aksy00@gmail.com; jcotas@gmail.com; leonel.pereira@uc.pt

Table S1. Statistical comparison of the experimental phases (p-value matrix of the ANOVA test)

		Phase 1	Phase 2	Phase 3
pH	Phase 1	-	<0.01	<0.01
	Phase 2	<0.01	-	0.051
	Phase 3	<0.01	0.051	-
Ammonium nitrogen	Phase 1	-	<0.01	<0.01
	Phase 2	<0.01	-	<0.01
	Phase 3	<0.01	<0.01	-
Nitrates	Phase 1	-	<0.01	<0.01
	Phase 2	<0.01	-	<0.01
	Phase 3	<0.01	<0.01	-
Orthophosphates	Phase 1	-	<0.01	0.015
	Phase 2	<0.01	-	<0.01
	Phase 3	0.015	<0.01	-
sCOD	Phase 1	-	<0.01	0.493
	Phase 2	<0.01	-	<0.01
	Phase 3	0.493	<0.01	-
CO_2	Phase 1	-	<0.01	<0.01
	Phase 2	<0.01	-	<0.01
	Phase 3	<0.01	<0.01	-

Table S2. Comparison of experimental procedure, treatment efficiency, and biomass valorization directions

Digestate type	Digestate Pretreatment	Microalgae inoculum	Reactor scale	Experimental conditions	Duration	Treatment performance	Biomass valorization	Refs.
liquid digestate from municipal wastewater treatment plant	without pretreatment	*T. obliquus*<br>*D. subspicatus*<br>*Microglena* sp.	20 dm^3	14 h light/10 h dark*<br>4800 Lux=68.57 $\mu mol/m^2/s$**	33 weeks	TN=96%<br>PO_4^{3-}=99%<br>sCOD=89%	towards lipids, fatty acids, pigments, proteins	This study
liquid digestate from vegetable waste	without pretreatment	*T.obliquus*<br>*D. subspicatus*<br>*Microglena* sp.	5 dm^3	14 h light/10 h dark*<br>3500 Lux=50 $\mu mol/m^2/s$**	15 weeks	TN=89%<br>PO_4^{3-}=73%<br>sCOD=91%	-	[17]
liquid digestate from vegetable waste	without pretreatment	*T. obliquus*<br>*D. subspicatus*<br>*Microglena* sp.	0.35 dm^3	14 h light/10 h dark*<br>2200 Lux=31.43 $\mu mol/m^2/s$**<br>↓	90 days	TN=71%<br>PO_4^{3-}=38%<br>sCOD=90%	-	[18]
				natural light and dark cycles*<br>5900 Lux=86.76 $\mu mol/m^2/s$**	90 days	TN=72%<br>PO_4^{3-}=66%<br>sCOD=84%		
				natural light and dark cycles*<br>5900 Lux=86.76 $\mu mol/m^2/s$**	90 days	TN=83%<br>PO_4^{3-}=88%<br>sCOD=93%		

liquid digestate from vegetable waste	without pretreatment	*T. obliquus*<br>*Microglena* sp.	0.35 dm^3	14 h light/10 h dark*<br>2200 Lux=31.43 µmol/m^2/s**	90 days	TN=70%<br>PO_4^{3-}=57%<br>sCOD=96%	-	[19]
		T. obliquus<br>*D. subspicatus*<br>*Microglena* sp.				TN=70%<br>PO_4^{3-}=43%<br>sCOD=94%		
piggery wastewater obtained from an anaerobic digestion	sedimentation<br>filtration<br>dilution<br>sterilization	*S. obliquus*	48 dm^3	12 h light/12 h dark*<br>200 µmol/m^2/s**	7 days	TN=58-75%<br>TP=70-89%<br>COD=62-75%	-	[52]
piggery wastewater and agricultural wastes	dilution	mainly *Chlorella* and *Scenedesmus* spp.	880 dm^3	natural conditions	200 days	TN=20±29%<br>COD=29±17%	-	[53]
liquid swine manure subjected to anaerobic digestion	dilution	*Chlorella vulgaris*	0.5 dm^3	continuous light*<br>50 µmol/m^2/s**	7 days	TN=67-71%<br>TP=49–54%<br>COD=74–79%	towards lipids, fatty acids, pigments, proteins, carbohydrate	[54]
	dilution<br>sterilization					TN=49–55%<br>TP=20–30%<br>COD=31–34%		
anaerobic digestate of thin stillage	dilution struvite recovery	*Chlorella sorokiniana*	1 dm^3	13 h light/11 h dark*<br>210 µmol/m^2/s**	18 days	TN=95%<br>TP=78%<br>COD=84%	towards proteins, starch, lipids	[55]
dairy manure digestate	dilution<br>filtration	*Chlorella* sp.	0.25 dm^3	continuous light*<br>200 µmol/m^2/s**	21 days	TN=76-83%<br>TP=63-75%<br>sCOD=27-38%	towards fatty acids	[56]
anaerobic digestate of dairy wastewater	without pretreatment	*C. sorokiniana*	0.2 dm^3	continuous light*<br>140 µmol/m^2/s**	10 days	TN=96±1%<br>TP=92±2%<br>COD=91±3%	towards carbohydrate, protein, lipids	[57]
anaerobic digestion effluent of food wastes	ozonation<br>autoclaving<br>pH adjustment	*Chlorella PY-ZU1*	0.3 dm^3	continuous light*<br>6000 Lux**	10 days	TAN=99%<br>TP=99%<br>COD=68%	towards carbohydrate, protein, lipids, fatty acids	[58]
anaerobic digestion effluent.	dilution	*Synechocystis* sp.	2 dm^3	continuous light*<br>200 µmol/m^2/s**	18 days	TN=100%<br>TP=100%	towards lipids, fatty acids	[59]

*Photoperiod
**Illumination intensity

Table S3. Statistical comparison of the BM1 and BM2 biomass (*p*-values of the t-test)

Compounds	Unit	*p*-value
Neoxanthin	%total pigments	0.256
Violaxanthin		0.244
Lutein		0.602
chla		0.051
chlb		0.790
β-carotene		0.170
chla	μg/mL	0.196
chlb		0.008
chla+chlb		0.032
Carotenoids		0.029
NLs	mg	0.042
GLs	mg	0.705
PLs	mg	0.069
Total lipids	mg	0.025
Proteins	% TS	0.005
